# The piloting of a culturally centered American Indian family prevention program: a CBPR partnership between Mescalero Apache and the University of New Mexico

**DOI:** 10.1186/s40985-017-0076-1

**Published:** 2017-12-22

**Authors:** Lorenda Belone, Ardena Orosco, Eloise Damon, Willymae Smith-McNeal, Rebecca Rae, Mingma L. Sherpa, Orrin B. Myers, Anslem O. Omeh, Nina Wallerstein

**Affiliations:** 1 College of Education, Department of Health, Exercise and Sports Sciences, Community Health Education Program, University of New Mexico, Albuquerque, NM USA; 2 Mescalero Apache Prevention Program, Mescalero Apache, NM USA; 3 Mescalero System of Care Program, Mescalero Apache, NM USA; 4 Center for Participatory Research, College of Population of Health, University of New Mexico Health Sciences Center, Albuquerque, NM USA; 5 Family and Community Medicine, University of New Mexico Health Sciences Center, Albuquerque, NM USA; 6 Public Health Program, Center for Participatory Research, College of Population Health, University of New Mexico Health Sciences Center, Albuquerque, NM USA

**Keywords:** American Indian, Indigenous, Community-based participatory research, Community-engaged research, Culturally centered, Health, Intervention

## Abstract

The Mescalero Apache Family Listening Program (MAFLP) is a culturally centered family prevention program with third, fourth, and fifth graders; a parent/caregiver; and a family elder. The program follows a positive youth development model to develop stronger communication and shared cultural practices between elders, parents, and youth in the tribe to reduce substance initiation of use among the youth. The MAFLP was created using a community-based participatory research (CBPR) approach in partnership with the University of New Mexico. The research focus of MAFLP is centered on the adaptation of a family curriculum from a Navajo and Pueblo version of the Family Listening Program to an Apache version, the establishment of a (Apache) Tribal Research Team, and the piloting of the curriculum with Apache families. MAFLP was piloted twice, and evaluation measures were collected focused on formative and impact evaluation. This article provides a background on Mescalero Apache then introduces the Navajo and Pueblo version of a Family Listening and Family Circle Program, respectively, next, the CBPR research partnership between Mescalero Apache and the University of New Mexico and the creation of a Mescalero Apache Tribal Research Team followed by the development and adaptation of a Mescalero Apache Family Listening Program including implementation and evaluation, and concluding with preliminary findings.

## Introduction

Substance abuse concerns have long plagued Native (American Indian/Alaska Native) communities, and health prevention approaches for Native children are in urgent need of attention. Existing mainstream programs fall short by failing to tailor culturally appropriate health messages and programs. Tribal communities have voiced a need for holistic programs that involve the whole family. The Mescalero Apache Family Listening Program (MAFLP) is a response to this request, and it integrates an evidence-based family-strengthening core, with Native cultural knowledge, history, values, and practices for late elementary-aged children and their parent/caregiver and family elder.

Over the past two decades, Tribal oversight and participation in research has grown to oppose historical abuses of research on Tribal communities which have resulted in negative stereotyping. Tribes have been pro-active in the creation of their own research policies, guidelines, and even institutional review boards with recognition of Tribal sovereignty. These new research policies have included the requirement that research benefit their communities, any data collected will be owned by the tribe, and all publications and/or presentations require tribal approval [1–5], including this manuscript. Tribal research principles have evolved and have included the right of Native communities to base research in their own knowledge and priorities, with participation in the research process requiring longer timelines, decolonized methodologies, and culturally centered interventions [2, 6–8]. There is growing evidence that culturally specific health interventions are highly successful and are being recognized by the Institute of Medicine [9–13]. However, some health interventions can be culturally superficial and seen as sufficient if all that is included is a cultural image, such as feathers or drums [14, 15]. In culturally centering an intervention, culture is not just seen as a set of beliefs or images but as people’s agency, voice, and power in the creation of the intervention while creating knowledge and reciprocal learning that can integrate culturally supported Indigenous practices and values [6, 16, 17].

New Mexico has a significant American Indian (AI) presence at 10.4% of the population, with rich historical traditions, including 23 Indian Tribes since time immemorial. According to the 2015 New Mexico Youth Risk and Resiliency Survey (YRRS) for high school students, Native youth in the state (~ 29% of the AI population are 17 years old and younger) reported several strengths: 64% plan to pursue higher education; 81% did not currently drink alcohol; and 84% did not currently use cigarettes. However, at-risk-behaviors were higher among Native youth as compared to the state: 33.8% marijuana use vs. 24.7% for the state; 17.9% used 2 or more illegal drugs vs. 11.7% for the state; and 34.9% used any tobacco product vs. 32.1% for the state [18].

Evidence shows that connection to history, land, language, traditional food, and culture have a positive impact on Indigenous health [19–21]. It is also shown that cultural connectedness is a protective factor against the negative impact of discrimination for AI adults [22]; and particularly for children, ages fifth to eighth grade, there is a positive impact on academic success [23, 24]. The Apache Family Listening Program uses cultural connectedness, empowerment, and a positive youth development model to delay substance abuse onset, reduce depressive symptoms, and enhance healthy behaviors. This research study has used a community-based participatory research (CBPR) approach which has been an appealing model for research with Native communities since it requires the involvement of the tribal community to be equally involved in all phases of the research study. In CBPR, a Tribal partner is engaged and are equal research partners, thus providing a promising approach in actively eliminating health disparities in Tribal communities [6, 25–29].

## Background

### Mescalero Apache

Mescalero Apache is located 3 h south of Albuquerque at the base of the Sacramento Mountains. The Mescalero Apache Tribe was established by Executive Order by President Ulysses S. Grant on May 29, 1873. The reservation became the central place for relocating three bands of Apaches—Chiricahua, Lipan, and Mescalero Apache. The Mescalero Apache homelands included the current reservation and greater Southwest regions of Texas, Arizona, and Mexico. The Lipan Apaches, whose original homelands spanned from Texas to Mexico, were relocated to Mescalero in the early 1900s. A couple hundred Chiricahua Apaches that were imprisoned at Fort Sill, Oklahoma, were moved to Mescalero around 1913. The Chiricahua Apaches homelands spanned throughout Arizona and Mexico. While the creation of the Mescalero Apache reservation is fairly recent (143 years), the histories of the three bands of Apaches span beyond hundreds of years. When the Tribe reorganized in 1936, the three bands became Mescalero Apache Tribal members, but families today still connect to their ancestral bands. The Mescalero Apache Reservation is approximately 720 mi^2^ and has more than 5000 enrolled members with a matrilineal-centered culture and language spoken as Southern Athabasca [30].

In 1965, the Mescalero Apache revised their tribal constitution, which established an election process for Tribal President, Vice-President, and Tribal Council. That same year, the late Wendell Chino was elected as the first Tribal President and held the position for 43 years by being re-elected 16 consecutive times. Under Mr. Chino’s presidency, the Mescalero Apache became a leader in many Native sovereignty issues including business enterprises, natural resources, and water rights. Mr. Chino was an advocate for tribal sovereignty and believed that Tribes had the right to make their own decisions regarding their government, education, land, business affairs, and economic development. In the 1970s, the Mescalero Apache did not renew contracts with the Bureau of Indian Affairs, so they could manage their own resources that included timber, grazing rights, mining, and water use. The Mescalero Apache fought for land, hunting, and fishing rights through the legal system and successfully secured the right to manage their own lands [31]. During this time, the Mescalero Apache also pursued various economic developments, most notably the development of the Inn of the Mountain Gods resort, which includes an 18-hole golf course and hotel. Other notable enterprises include the Ski Apache resort located on the sacred Sierra Blanca Mountain, the Mescalero Apache Tribal Store, and the Mescalero Apache Travel Stop [32].

In addition to securing land rights and successful economic development, Mr. Chino fought to secure funding to develop a K–12 school on the reservation. Prior to 1995, only an elementary school and middle school existed on the reservation. Mescalero Apache high-school students had to commute to two neighboring non-Native towns (30-min drive north or south) to attend these high schools. The Mescalero Apache K–12 School graduated its first class in 1996. Even though Mescalero Apache has a variety of well-established alcohol and medical treatment programs, the MAFLP was the first prevention program that focused on elementary-aged students and their parent/care giver.

### Family Listening/Family Circle Program (FLCP)

In 2000, the Native American Research Center for Health (NARCH) was established by the Indian Health Service and the National Institutes of Health (NIH) as a national American Indian/Alaska Native (AI/AN) research funding mechanism to reduce AI/AN health disparities, address the distrust of research by AI/AN communities, and support a pipeline for AI/AN researchers. The NARCH grant application process differs from the NIH process based on the fact that a NARCH application is submitted by a Tribe or inter-Tribal organization who then partners or subcontracts with a research institution [6, 33].

Utilizing the NARCH mechanism, the Pueblo of Jemez [6] and the Ramah Band of Navajo [26] partnered with the University of New Mexico Center for Participatory Research (UNM-CPR) based on the learnings and consultations from Dr. Whitbeck at the University of Nebraska and his Anishinabe partners who developed an intervention called the *Bii-Zin-Da-De-Dah* program*,* translated as *Listening to Each Other*, funded by NIH*.* The program produced evidence of effectiveness as a psychosocial, cultural, educational intervention, resulting in a curriculum that combined both cultural messages and mainstream parenting communication skills. The *Bii-Zin-Da-De-Dah* found that the Anishinabe youth were better able to retain prevention messages if they were culturally embedded [6, 22].

**Fig. 1:**
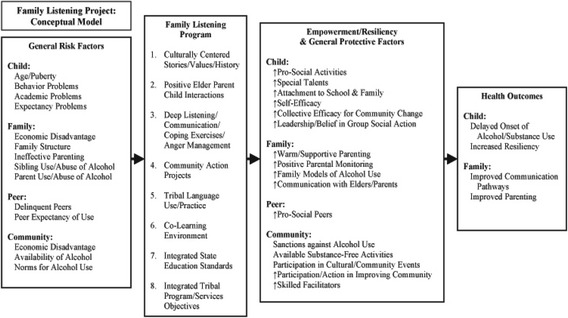
Family Listening Program Conceptual Model

The Pueblo, Navajo, and UNM-CPR partnership received a NARCH III research grant (2005–2009) to adapt the *Bii-Zin-Da-De-Dah* program while utilizing a CBPR approach to develop a culturally centered Navajo and Pueblo curriculum, called Family Listening and Family Circle (FLCP), respectively. The program curriculum addressed literature on risk factors and integrated evidence-based theories such as change with cultural practices and values to support child, family, and community resiliencies and empowerment, including parent-child communication, having adult mentors and support, and empowerment strategies. By recognizing and addressing these risk factors, FLCP is a preventative approach to increase resiliency and delay substance use among third, fourth, and fifth graders as shown in the conceptual model (Fig. 1).

### Case presentation Mescalero Apache and UNM-CPR research partnership

During the NARCH III study, UNM-CPR presented on the Navajo FLP research project at an Albuquerque Area Indian Health Board (AAIHB) advisory meeting which at the time had research oversight through an administrative core of NARCH III. During this presentation, one of the AAIHB advisory members from Mescalero Apache heard about the program and requested that FLP also be brought to Mescalero Apache. In being true to the CBPR process, the initial request came from the community in the form of a resolution from the Mescalero Apache Tribal Council for UNM-CPR and Mescalero Apache to co-write and co-submit a NARCH V grant application. In 2009, Mescalero Apache and UNM-CPR were awarded NARCH V funding to establish a research partnership. The aim of the study was to utilize a CBPR approach to establish a Mescalero Apache Tribal Research Team to be trained in a CBPR research approach, to co-adapt an intergenerational family prevention curriculum for late elementary-aged youth and their families based on Mescalero Apache culture and values, and pilot the adapted curriculum with ten families. Over the course of this study, human research protection was obtained from the UNM Human Research Protection Office (HRPO-11-217) and the Southwest Tribal Institutional Review Board (SWT-2011-005) housed within the Albuquerque Area Indian Health Board.

### Tribal Research Team

Recruitment of tribal members to serve on a research community advisory committee (CAC) in partnership with UNM-CPR was conducted in year one of the NARCH V research study, and continued relationship building and on-going recruitment occurred throughout the grant years. Community members who worked with children, youth, parents, and elders’ prevention programs were recruited from the following programs: Tribal Community Services Committee, Rehabilitation Center (Alcohol/Drug Addictions Treatment Center), Behavioral Health, the Indian Health Service Hospital, Mescalero Apache Housing Authority, Bureau of Indian Education (BIE) School, and the Boys and Girls Club. The CAC slowly came together with varying membership and participation from four to ten members to monthly meetings over the course of the study. Several trainings were conducted with the CAC and always in their community, such as public health 101, research 101, focus group facilitation, visioning facilitation, institutional review board (IRB) confidentiality, data collection/data analysis/interpretation training, and principles of participatory research. Based on the fact that the CAC had gained extensive research knowledge and skills, the UNM-CPR viewed them as a skilled research team and changed their title to Tribal Research Team (TRT). The first document developed in partnership between the TRT and UNM was a set of mutually agreed-upon principles of research team participation and roles and responsibilities with monthly meetings. This agreement and the roles and responsibilities were revised yearly but revisions were not required in the final grant years.

### Family curriculum development

Development of the MAFLP curriculum involved the initial review of the Pueblo and Navajo curriculum which were 14 sessions. Upon completion of initial review, the TRT planned and conducted focus groups with service providers, youth, parents, and elders around cultural aspects of the curriculum so that Mescalero Apache history and values could be included into a new Apache family curriculum. Sections from the Pueblo/Navajo curricula that required minimal modifications were the sessions with evidence-based findings, such as the sessions on anger management, parent communication skills, and help-seeking behavior. Some of these sessions were merged to pare down the Apache curriculum from 14 sessions to 12 since the TRT felt 14 weeks would be a challenge to sustain active involvement by families. While the sessions (see Table 1) in the curriculum were scaled down, the TRT felt it was important to expand the history session from one to two sessions, due to the importance of providing enough time for dialog. This was also a lesson learned from the Pueblo/Navajo study.

**Table 1:** Navajo, Pueblo, and Apache sessions of the Family Listening/Circle Program

Sessions	Navajo curriculum	Pueblo curriculum	Mescalero Apache curriculum
1	Welcoming	Welcoming	Welcoming
2	My family	Family dinner	Apache history (part I)
3	Navajo history	Pueblo history	Apache history (part II)
4	Navajo way of life	Pueblo way of life	My family
5	Our Navajo vision	Our Pueblo vision	Apache way of life
6	Community challenges	Community challenges	Apache vision
7	Community and help seeking	Communication and help seeking	Community challenges
8	Recognizing types of anger	Recognizing types of anger	Communication, help seeking, and problem solving
9	Managing anger	Anger management	Recognizing types of anger and managing anger
10	Problem solving	Problem solving	Being different and positive relationships
11	Being different	Being different	Building social support
12	Positive relationships	Positive relationships	Making a commitment and community project presentations
13	Building social support	Building social support	
14	Making a commitment and CAP presentations	Making a commitment and CAP presentations	

Language preservation was an important component by the TRT who shared that the participating youth may have more Apache language knowledge than their parents based on the fact that the youth were attending Apache language classes at the tribal school. Recognizing the potential difference in language knowledge, the TRT reached out to the Mescalero Apache Language Program who assisted in the creation of conversational flash cards, 26 in total, with Apache and English interpretations that were utilized during each session. The flash cards included 13 short conversations where a question would be asked and a response would be given, for example “how are you?” “With me, it is good”; “where are you coming from?” “From my house”; and “When did you get here?” “Just a while ago”. The creation of the flashcards was a new component of the FLP program; however, the curriculum format remained consistent with the Pueblo/Navajo versions. The Apache adaptations centered on Apache culture through images and historical photos which replaced any Pueblo/Navajo information throughout the curriculum. The images were produced by a local Apache artist who participated in several research meetings and based on his interest created images to be added to the curriculum. The artist produced several beautiful paintings reflecting Mescalero Apache culture that centered on family, history, and ways of traditional life. In addition to these images, several historical photos depicting Mescalero Apache life from over 100 years prior were provided by the tribal cultural preservation office to also be included in the curriculum.

### Piloting of family intervention

Once the MAFLP family curriculum was finalized, the TRT recruited third, fourth, and fifth graders and their parents/care givers to participate in the piloting of the newly created family program. Facilitators of the program were also recruited, first from the TRT and then from the greater community. The main facilitators were individuals from the Mescalero Prevention Program and additional facilitators were from other Tribal programs who were trained in the context and delivery of the family curriculum with a total of six trained tribal facilitators. Each of the 12 sessions required four facilitators primarily so that two facilitators would be available when the children and the adults broke into separate groups for an age-appropriate activity and dialogue. Generally, each session was held in the evening and started with a prayer usually by a child participant and in their Native language; the Apache flash cards were then practiced with the participants and facilitators standing in a circle, based on the card a conversational question would be asked in Apache to the person on the left and the question was then answered in Apache; this was repeated until all participated in the circle; dinner then followed allowing families and participants to share a meal while getting to know one another. After dinner, the participants shared their home practice, an activity sheet given in the prior session and completed at home; an “ice breaker” activity then followed, allowing families to engage in a fun activity as a way to build relationships. Next, the adults and the children separated into separate rooms to focus on the session topic, at the end of the session the adults and the youth were given time to journal and to reflect on the session. The evening for the families would come to an end by the handing out of a small incentive usually imprinted with the MAFLP logo (e.g., drawstring bags, photo keeper, lunch bags, etc.), and each facilitator would then complete a session evaluation. The family program continued this process over 11 sessions with the final session centered on families presenting their community action projects. The 12 sessions continued over 5 months, beginning in the fall and ending in early spring.

### Evaluation design

All the instruments that were utilized were co-developed with the Navajo/Pueblo TRTs in the Navajo/Pueblo pilot [6, 26], the measures collected formative and impact findings of the MAFLP. The goal of the formative evaluation was to measure and document program implementation feasibility, while the goal of the impact evaluation was to measure the preliminary outcome of the program in improving cultural connectedness, family communication, and coping skills while reducing anxiety and depression and risky behaviors among the program participants. The MAFLP was piloted twice in this study. The first pilot was initiated June through July 2013, 2 days a week, with 12 children and 7 adults. The second pilot ran from January to May 2014, 1 day a week, with 9 children and 10 adults.

### Quantitative data

All participants completed a baseline survey prior to their participation in the program and a follow-up survey immediately at the completion of the program; these questions were closed-ended. The follow-up survey included three additional questions which were open-ended. The child survey included questions around demographics, school safety, substance use, general health, home life, self-efficacy, social support, anxiety, and Apache culture. The adult survey included demographics, housing and reservation life, social support, traditional activities and culture, spirituality, identity and language, historical loss, discrimination, coping strategies, community problems, substance use, and parenting skills. The one difference between the child/adult baseline and follow-up surveys were the inclusion of three qualitative questions which centered on observational changes, for example (a) have you seen any changes in your family since participating in this program?; (b) have you seen any changes in your behavior since participating in this program?; and (c) have you seen any changes in your child/parent since participating in this program? Participants completed a paper format of the survey which was entered into SPSS for additional data management and analyses. SAS v9.3 was also used for data management and analyses. Quantitative data were Likert-type measures or categorical variables. Baseline and follow-up comparisons of continuous variables were analyzed using two-sample nonparametric Wilcoxon tests as our tribal IRB specified that identities not be tracked. Means and standard deviations are reported for continuous variables, and frequency and percent are used to summarize categorical variables.

### Qualitative data

The qualitative measures included attendance sheets, facilitator logs, and observations of the facilitators by the UNM team. At the beginning of each of the 12 sessions, the participants signed in; the sheets helped to assess dose. At the completion of each session, the facilitators completed a facilitator evaluation form reflecting on their role as a facilitator with questions specific to the session topic (see Table 2). UNM observations included fidelity to the curriculum objectives, flow of delivery, and barriers to program implementation. The UNM team attended all the sessions as observers and provided assistance to the facilitators as needed.

**Table 2:** Formative evaluation

Goal	Process measures	Process outcomes
Program fidelity	• Class attendance • Facilitator logs	• Participants enjoyed the activities in the sessions • Participants were willing to learn • Finding ways to help participants overcome being shy • Ways to encourage participants to return to next session • Providing more time to think • Ways to work with participants to help them talk more

Additional qualitative measures included the examination of weekly journals by the participants who reported on their experiences and reflection after each session of the program. Each journaling session asked the questions “What two things did you learn from this session’s activities and discussions?” All weekly journals were collected and transcribed and analyzed for themes.

## Results

### Quantitative findings

Twenty-one children and 17 adults participated in the study (see Table 3). All the adults completed the baseline survey and only 11 (65%) completed the follow-up survey. The adults were mostly women (76%) who were single (62%). About 90% of the adults who completed the three qualitative follow-up questions reported seeing a change in their family after participating in the program. Of this 90%, 82% reported seeing changes in themselves and changes in their child.

**Table 3:** Characteristics. Mescalero Family Listening/Circle Program pilot project participants, 2013–2014

		Baseline	Follow-up
Age group	Characteristic	*N* (%)	*N* (%)
Child	All	21	11
	Gender female	13 (62)	7 (64)
	Grade		
	Third	6 (30)	0 (0)
	Fourth	8 (40)	6 (60)
	Fifth	6 (30)	4 (40)
Adult	All	17	11
	Gender female	13 (76)	8 (73)
	Age, median (min, max)	41 (29, 59)	34 (29, 60)
	Marital status		
	Married	4 (25)	2 (18)
	Single	10 (62)	3 (27)
	Living with partner	2 (12)	5 (45)
	Employment		
	Full time	5 (31)	4 (44)
	Part time	1 (6)	3 (33)
	Self-employed	2 (12)	0 (0)
	High school graduate or GED	11 (65)	9 (82)

Twenty-one children completed the baseline survey and only 11 (52%) completed the follow-up survey. Sixty-three percent of children who completed the baseline survey were female with a majority of them in the fourth grade (40%). Of the children participating in the follow-up survey, seven (64%) reported seeing changes in their family after participating in the program, and six (55%) reported seeing changes in their behavior. Additionally, 70% (*n* = 6) of the children answered that they had seen changes in their parents since participating in the program. On the problem-solving scale, the participants reported an increase in their problem-solving skills, such as working out their problems by talking or writing about them (baseline mean = 2.15, SD = 1.18; follow-up mean = 3.09, SD = 1.22, *p* = 0.063). The participants also recorded a significant impact on the culture scale: for example, a question asking if the child saw themselves as Apache reported a significant increase after program participation (baseline mean = 4.10, SD = 1.00; follow-up mean = 4.82, SD = 0.40, *p* = 0.015). On the depression and anxiety scale, participants reported feeling less restless and on edge upon program completion (baseline mean = 2.05, SD = 1.07; follow-up mean = 1.30, SD = 0.67, *p* = 0.049). The program participants recorded a significant positive change on the parenting question, which said “I punish child by taking privileges away with little if any explanation” (baseline mean = 3.06, SD = 1.39; follow-up mean = 1.92, SD = 1.08, *p* = 0.037).

### Qualitative findings

The facilitator logs were analyzed for themes, and a common theme found across all the sessions was that the facilitators enjoyed the session activities and that the participants were willing and eager to learn. A recommendation by the facilitators based on the logs noted that sessions could be improved through finding ways to encourage the participants to overcome shyness and finding ways to encourage the participants to return to the next session, such as personal follow-up either through a phone call or a home visit. The facilitators were also able to apply problem-solving strategies to every issue that arose during each session. An important theme focused on self-reflection and the fact that the facilitators shared a sense of increased confidence in their facilitation skills as the program continued. The observation by the UNM team provided opportunities to update the curriculum based on the facilitators’ recommendation, modifications to the flow of the session, and any other changes that were made to the program.

A major finding from the journals from the child and adult participants reported having new learnings from each of the sessions. One child participant stated “I learnt how to trust and support”. One adult participant stated “I learnt that it is okay to be angry, but how you deal with it depends on you. Anger issues can be worked through discussing the issue that made you angry”. The children also reflected on learning new skills, such as problem solving, ways of listening, anger control, and ways of supporting their community. An area of concern raised by the children focused on the outlook of the community, particularly on substance use and abuse. Some parents shared similar concerns but also added concerns of lack of housing and alcohol use and abuse.

## Discussion

The MAFLP was intended to focus on children; however, this unique program provided a whole family participation format, allowing parents and elders to experience this program alongside their child while learning skill-building exercises around communication, listening, anger management, respecting diversity, and building trust.

The Mescalero Apache TRT and facilitators were pleased with the two pilots and the level of involvement by the participants in the session topics, session activities, and small group discussions. Based on the first pilot, it became apparent that there was a need to reach out to the participants and to remind the families of the upcoming session. In the second pilot, the facilitators were more active in reminding the families and felt that this was a positive change in the second pilot. Overall, the adult participants were pleased that their child learnt communication and problem-solving skills but more importantly he/she learnt the importance of being respectful, the Apache stages of life, ways of being helpful to the community, and the Apache history.

Although this pilot study had a very small sample size, we were able to observe significant positive changes in problem-solving skills, cultural knowledge, depression and anxiety, and parenting skills. Positive coping skills and a strong cultural identity have been identified as protective factors against drugs and alcohol use as children move into adolescence. While we saw no difference in substance use, we believe this was based on the fact that the program only ran for 5 months from baseline to follow-up; we believe a second follow-up would have been beneficial. Fortunately, we have continued this research partnership with Mescalero Apache in a current R01 study that includes a longitudinal design with a comparison group.

## Conclusions

The Mescalero Apache and the University of New Mexico research teams successfully collaborated in a CBPR approach to create and twice pilot a culturally centered Mescalero Apache Family Listening Program based on the adaptation and lesson learnt from a Pueblo and Navajo version. The MAFLP child participants were able to increase their problem-solving skills and cultural identity while reducing depression and anxiety. The results of the pilot evaluation were promising although the long-term impact of the intervention remains to be seen and requires further evaluation. Our pilot findings provided us with data on fidelity and dose of intervention, satisfaction, and acceptability of the program by participants and feedback on areas of improvement. Based on the piloting of the FLCP, the three Tribal communities [6, 26] were eager to continue the research partnership with UNM and were successful in obtaining a National Institute on Drug Abuse R01-funded study (2014–2019) to rigorously test the effectiveness of the three American Indian family programs with a comparative longitudinal design within and across the three Tribal communities with fourth and fifth graders to prevent substance abuse initiation disparities through the strengthening of family well-being and self-identity.

The R01 study design includes a two-arm non-equivalent control group with baseline, immediate follow-up, and a 1-year follow-up. With this design, we will be able to analyze the impact of the FLCP intervention between the program participants and a comparison group aggregated across the three Tribal communities through four waves of implementation. We will also be able to analyze the diffusion of the program over time. Based on the pilot studies [6, 26], the three TRTs gained vital research experience in recruiting participants, administering the baseline and follow-up surveys, and implementation of the program and are prime as co-research members in the R01 study. In CBPR, the community is an equal research partner allowing for active engagement in addressing a health disparity [29]. The R01 study utilizes a CBPR approach with Tribal communities who are equal research partners and therefore are posed in actively addressing the health disparities of substance initiation of use through a culturally centered family strengthening program focused on increased cultural knowledge and identity.

## Abbreviations

AAIHB: Albuquerque Area Indian Health Board
AI: American Indian
AI/AN: American Indian/Alaska Native
CAC: Community advisory committee
CAP: Community action project
CBPR: Community-based participatory research
FLCP: Family Listening/Circle Program
FLP: Family Listening Program
MAFLP: Mescalero Apache Family Listening Program
NARCH: Native American Research Center for Health
TRT: Tribal Research Team
UNM-CPR: University of New Mexico Center for Participatory Research
